# Population Genetic Analysis of Ten Geographically Isolated Tibetan Pig Populations

**DOI:** 10.3390/ani10081297

**Published:** 2020-07-29

**Authors:** Peng Shang, Wenting Li, Zhankun Tan, Jian Zhang, Shixiong Dong, Kejun Wang, Yangzom Chamba

**Affiliations:** 1 Animal Science College, Tibet Agriculture and Animal Husbandry University, Linzhi 860000, China; nemoshpmh@126.com (P.S.); tanzhankun@xza.edu.cn (Z.T.); zhj88n@126.com (J.Z.); herko1@163.com (S.D.); 2 College of Animal Sciences and Veterinary Medicine, Henan Agricultural University, Zhengzhou 450001, China; liwenting_5959@hotmail.com

**Keywords:** domestication, selection, conservation, genetic diversity

## Abstract

**Simple Summary:**

Whole-genome re-sequencing data from 10 geographically isolated Tibetan pig populations were collected and analyzed in this study. Population genetic analyses, including Principal Component Analysis (PCA), phylogenic tree, genetic differentiation, deleterious variant, contribution to meta-population genetic diversity and selective sweep were performed. Limited genetic differentiation was identified among these Tibetan pig populations. Most deleterious variants were low-frequency mutations and population specific. Contribution to the meta-population was largest in the TT population, based on gene and allelic diversity. Genes under selection were involved in hypoxia adaptation, hard palate development, facial appearance, and perception of smell.

**Abstract:**

Several geographically isolated populations of Tibetan pigs inhabit the high-altitude environment of the Tibetan Plateau. Their genetic relationships, contribution to the pool of genetic diversity, and their origin of domestication are unclear. In this study, whole-genome re-sequencing data from 10 geographically isolated Tibetan pig populations were collected and analyzed. Population genetic analyses revealed limited genetic differentiation among the Tibetan pig populations. Evidence from deleterious variant analysis indicated that population-specific deleterious variants were the major component of all mutational loci. Contribution to the meta-population was largest in the TT (Qinghai-Tibet Plateau) population, based on gene diversity or allelic diversity. Selective sweep analysis revealed numerous genes, including RXFP1, FZD1, OR1F1, TBX19, MSTN, ESR1, MC1R, HIF3A, and EGLN2 which are involved in lung development, hard palate development, coat color, hormone metabolism, facial appearance, and perception of smell. These findings increase our understanding of the origins and domestication of the Tibetan pig, and help optimize the strategy for their conservation.

## 1. Introduction

Tibetan pigs originated on the Tibetan Plateau, and the record of their breeding by humans can be traced back to the 7th century [1]. Tibetan pigs are adapted to hypoxia, and a low temperature, low-pressure environment due to long-term natural selection [2,3,4,5]. Considerable research has focused on their origins, physiological features, and adaptation to their high-altitude environment. mtDNA evidence has revealed the origin centers of the Tibetan pig [6], and nuclear genome analysis has shown that the Tibetan pigs originated exclusively on the Tibetan plateau and survived in a wild state [7]. Results from SNP-chip data showed that the Tibetan pig was a domestic indigenous pig living on the Tibetan plateau and underwent genetic differentiation among three populations [4]. 

In this study, using whole-genome resequencing data, we analyzed the genetic relationship among the 10 geographically isolated Tibetan pig populations as well as between Tibetan pigs and wild pigs. Gene and allelic diversity of each Tibetan pig population was calculated to identify their contributions to the population pool. Finally, selective signatures in the genomes of Tibetan pigs were scanned to identify the genes involved in adaptation to the harsh environment of the Tibetan plateau. 

## 2. Materials and Methods

### 2.1. Sample Collection

A total of fifty Tibetan pigs from 10 geographically isolated populations and 15 wild pigs were used for this study. As reported in a previous study, all Tibetan pigs were collected from the Qinghai-Tibet Plateau, including Chinese Tibet, Sichuan, Yunnan, and Gansu province [7,8]. As described in Mingzhou Li et al. and Huashui Ai et al. the populations are genetically unrelated, having no common ancestors within three generations [7,8]. The raw data of whole-genome resequencing was downloaded from the Sequence Read Archive (NCBI accession number available in Table S1). Detailed information on the Tibetan pig populations is shown in Table 1 and Table S1. All animals were handled, housed, and all procedures were performed following the guidelines approved by the College of Agriculture and Animal Husbandry, Tibet University.

### 2.2. Read Mapping and SNP Calling

Libraries with inserts of 500 bp were constructed for each pig. 100-bp paired-end reads were generated using the HiSeq2000 platform [7,8]. Raw reads were filtered (seed mismatches, palindromes simple clip—2:30:10) using Trimmomatic [9]. The BWA-MEM program, with the default parameter settings, [10] was used to map the filtered reads against the reference genome (Ensemble *Sscrofa11.1*). SAMtools (v1.9) was used to merge and sort the BAM files [11]. Duplicate reads were marked using MarkDuplicates from Picard-Tools (http://broadinstitute.github.io/picard/). Subsequently, SNP calling was performed for each individual pig using GATK4 HaplotypeCaller then joined using the GenomicsDBImport and GenotypeGVCFs programs from GATK4 (v4.1.2.0) [12]. To get a reliable dataset, the VariantFiltration program from GATK4 was used to filter the SNPs with the parameters “*QD < 2.0 || FS > 200.0 || SOR > 10.0 || MQRankSum < −12.5 || ReadPosRankSum < −8.0*”. 

### 2.3. Phylogenic Tree Construction and PCA

Using VCFtools (0.1.16), biallelic SNPs located on autosomes were identified and kept (http://vcftools.github.io/) [13]. Using PLINK(v1.9) [14], variants with a call rate >90% and MAF > 0.05 were identified and retained (http://www.cog-genomics.org/plink2/). Before constructing the phylogenetic tree, high-density SNPs were pruned using PLINK (v1.9) with the parameters “--indep-pairwise 50 5 0.2”. The phylogenetic tree was constructed using the IQ-TREE software (multicore version 1.6.12) with 1000 bootstrap iterations based on the PHYLIP format data [15]. The phylogenetic tree was visualized using the iTOL web server [16]. Principal Component Analysis (PCA) was also performed with pruned data using the SMARTPCA program of EIGENSOFT [17].

### 2.4. Genetic Diversity and Deleterious Variants

Expected heterozygosity (H_E_), observed heterozygosity (H_O_), and the inbreeding coefficient for each Tibetan population were estimated using PLINK (v1.9). Nei’s minimum genetic distance and the gene frequency differentiation index (F_ST_) between pairs of subpopulations were computed using Metapop v2 [18]. Ensembl Variant Effect Predictor tool (VEP, v98.3) was used to assess the deleterious variants across autosomes. It is well-known that the SIFT score of VEP is sensitive to the alleles that are likely to be predicted as deleterious. Variant loci with a SIFT score close to 0 were considered deleterious [19]. Using the R UpsetR package [20] their distribution and intersecting sets among populations were analyzed and visualized. 

To estimate the maximal contribution to heterozygosity of each population, total heterozygosity was partitioned into the average expected heterozygosity within subpopulations (H_S_) and average Nei’s minimum genetic distance between subpopulations (D_G_) [21]. Similarly, total allelic diversity (A_T_) was partitioned within and between the subpopulations. Average allelic diversity within subpopulations is calculated as the average number of alleles segregating in the subpopulations minus one (A_S_), while average allelic diversity between subpopulations is calculated as the average number of unique alleles in a subpopulation compared to the other subpopulations averaged over all possible subpopulation pairs (D_A_) [18]. Using the Metapop2 program, the above-mentioned indicators were used to estimate the expected contribution of each subpopulation to gene and allelic diversity of the meta-population by removing the subpopulation from the analysis and computing the change in diversity [18]. 

### 2.5. Selective Sweeps

Selective sweeps were scanned with sequence diversity statistic *θ*_π_ [22] and population differentiation statistics *F_ST,_* using a 10 kb no-overlapping window. To identify genomic regions under selection in Tibetan pigs, genome-wide *θ*_π_ and *F_ST_* were collected. Z-transformed *F_ST_* and *θ*_π_ ratios were analyzed using the scale function included with the R package. Genomic signatures were defined by a high Z-*F_ST_* (Top 1%) and Z-*θ*_π_ ratio (*θ*_π,wild_/*θ_π_*_,Tibetan_, Top 1%). Genes located in the selective signature were obtained using R ‘biomaRt’ package [23], and then subjected to GO enrichment analysis using the Panther web server (http://www.pantherdb.org).

## 3. Results and Discussion

In this study, sequencing data from 50 Tibetan pigs, from 10 geographically isolated populations of the Qinghai-Tibet Plateau, and 15 wild pigs were collected and analyzed (Table 1 and Table S1). Approximately 47.4 million autosomal biallelic SNPs were ultimately identified. 

### 3.1. Population Structure Analysis

**Figure 1:**
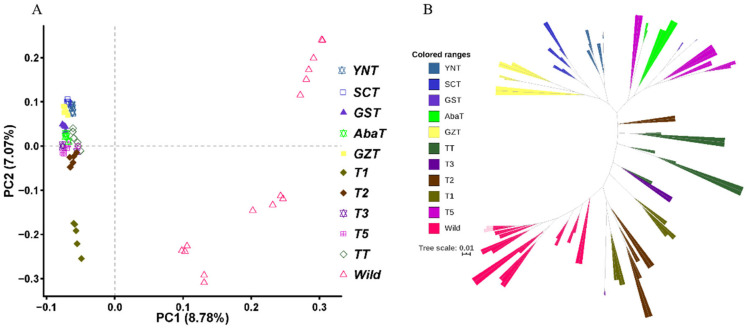
Principal component analysis (**A**) and the phylogenic tree (**B**) of 10 geographically isolated Tibetan pig populations.

To investigate the genetic structure of 10 Tibetan pig populations and their relationship to wild pigs, we utilized principal component and phylogenetic analysis. For the first principal component (8.78%), no obvious segregation was observed among Tibetan pig populations, while significant differentiation was shown between Tibetan pig and wild pig populations (Figure 1A). Evidence from the second principal component (7.07%) demonstrated that the T1 population was significantly differentiated from others, and the GZT, SCT, and YNT populations were slightly differentiated from the others. These data suggest that genetic drift or blood mixture occurred in the T1 population. Similar results were also observed in the phylogenetic relationships, namely the GZT, SCT, and YNT populations formed a small clade, while T1 and T2 were more like wild pigs (Figure 1B). This phylogenetic pattern was similar to that reported in a previous study based on SNP chip data [4]. In that study Tibetan pigs from Yunnan and Sichuan province were grouped and found to be closer to geographic neighbors’ other local breeds. The Ancient Tea Horse Road was a trade route between Tibet and Southeastern China for over 2000 years. Ancient Tea Horse Road, including the Yunnan-Tibet and Sichuan-Tibet routes, enabled commodity exchange between Tibet and Yunnan and Sichuan provinces and likely played an important role in the modern genetic makeup of Tibetan pigs [4]. Analysis of mitochondrial DNA from Tibetan pigs has provided a local origin of domestication [6], and suggests that genetic differentiation of the 10 Tibetan pig populations may be due to breed mixing from other ancient indigenous pigs or genetic drift in the current small population.

### 3.2. Genetic Diversity and Differentiation

**Figure 2:**
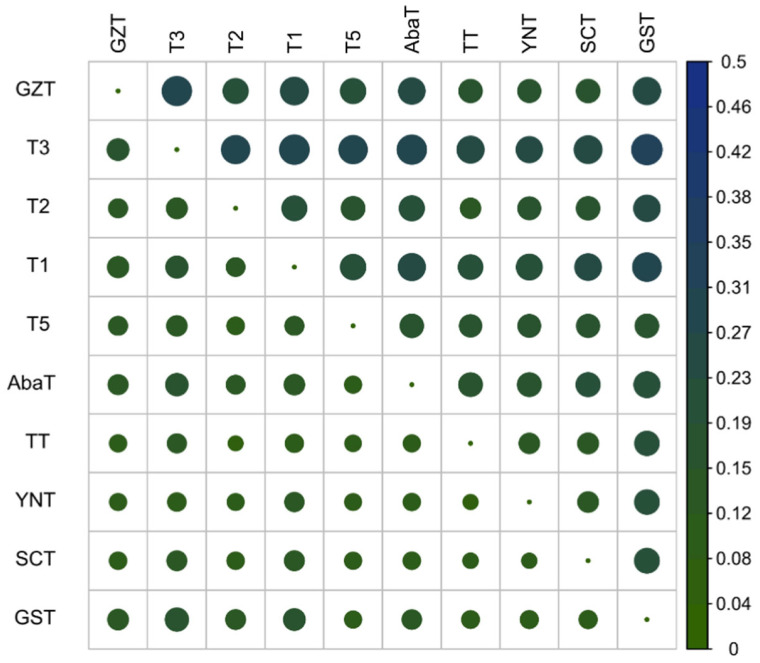
Heatmap plot of Nei’s minimum genetic distance *D_nei_* (**upper**) and gene frequency differentiation index *F*_ST_ (**lower**).

Pair-wise genetic differentiation index (*F*_ST_) and pair-wise Nei’s minimum genetic distance (*D_nei_*) were examined among the Tibetan pig populations (Figure 2). Lower differentiation values were found among GZT, SCT, and YNT populations based on *F*_ST_ (0.081, 0.1024 and 0.1066) and *D_nei_* (0.1499, 0.1885 and 0.1952). The greatest differentiation was observed between the GST and T3 populations based on *F*_ST_ (0.1904) and *D_nei_* (0.3261), while the least differentiation was between T2 and TT, based on *F*_ST_ (0.0775) and between TT and YNT based on *D_nei_* (0.1473). The smallest averages of differentiation and genetic distance were between TT and the other populations (average *F*_ST_, 0.0999; average *D_nei_*, 0.1880). This result is consistent with the population structure, as the TT population is located in a relatively central position of the PCA and phylogenic tree (Figure 1). The largest averages of differentiation and genetic distance were between T3 and the other populations (average *F*_ST_, 0.1545; average *D_nei_*, 0.2869). The second-largest averages of *F*_ST_ and *D_nei_* were between T1 and the other populations (average *F_ST_*, 0.1421; average *D_nei_*, 0.2522). Collectively, these results are consistent with the population structure. 

Genetic diversity (*Ho*, *H_E_*, *A_R,_* and *F)* was calculated for each population based on whole-genome biallelic SNPs (Table 1). The range of values by each measure was 0.2379–0.4385 (*Ho*), 0.3678–0.4178 (*H_E_*) and 1.4769–1.6028 (*A_R_*). The maximum *Ho* was in the GST population (0.4385), followed by T3 (0.4036), and the minimum was in the T5 population (0.2379). The maximum *H_E_* was in the T3 population (0.4682), followed by AbaT (0.4178), and the minimum was in the TT population (0.3670). Some of the differences observed between *H_E_* and *H_O_* are due to different levels of inbreeding in each population. To assess the level of inbreeding within a population, average inbreeding coefficients for each were calculated. The range of inbreeding coefficients was 0.0083 (YNT) to 0.3537 (T5) (Table 1). The greatest inbreeding coefficient and lowest heterozygosity were observed in the T5 population. Although estimation of these indicators can be biased due to the small population size, whole-genome SNP data from unrelated samples would reduce this bias to some extent.

### 3.3. Deleterious Variants and Their Contribution to Gene and Allele Diversity

**Figure 3:**
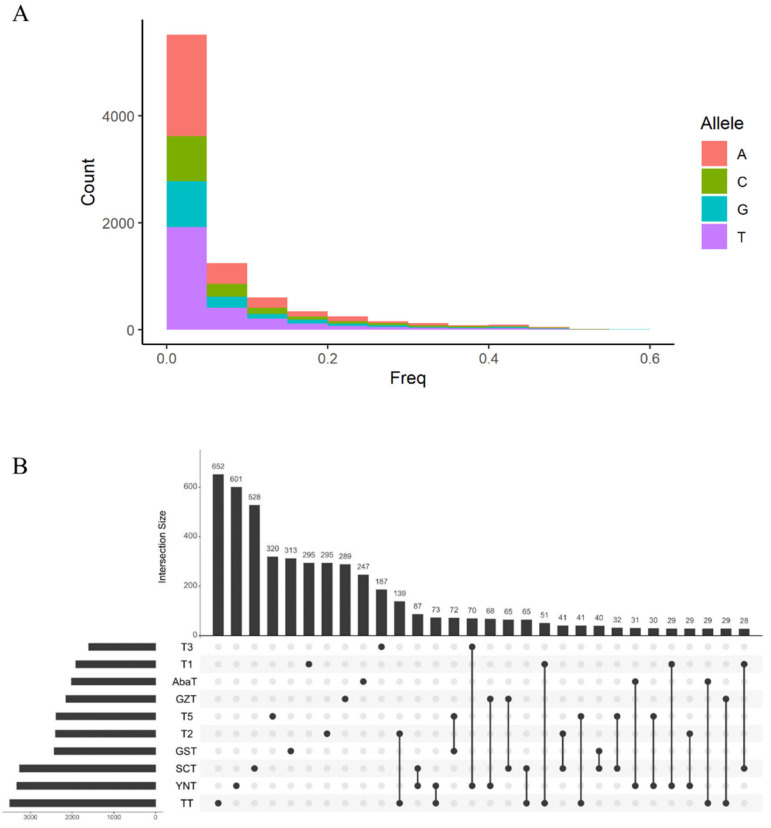
Distribution of deleterious variants. The frequency spectrum of deleterious variants (**A**). Intersect plot of deleterious variant among 10 Tibetan pig populations (**B**).

Conservation management aims to maintain genetic diversity to maximize survival potential and fitness [19]. Often deleterious variants are used to estimate the fitness of a population. Here we used the Ensembl Variant Effect Predictor to identify putative damaging mutations across the genome; variants with a SIFT score close to 0 were designated as such, and a total of 8490 putative deleterious variants were identified. The frequency distribution of deleterious variants is presented in Figure 3A and Table S2, most are low-frequency mutations, ranging from 0 to 0.59. Allele frequency of over half the deleterious variants was 0 to 0.05 and the majority of mutations were purine to pyrimidine bases (Figure 3A). Using whole-genome resequencing data, Bosse et al. identified 3129 and 3468 low-frequency putative deleterious variants in *Sus cebifrons* and *Pietrain* pig populations respectively [19]. Similarly, we found that the number of deleterious variants varied among the Tibetan pig populations, ranging from 1606 (T3) to 3499 (TT); only 229 of these were present in all populations (Figure 3B and Table S2). Most deleterious variants were population-specific, which ranged from 652 (TT) to 187 (T3) (Figure 3B). Furthermore, the pair-wise variant number of intersection sets were all less than 100 except for the T2-TT pair. Subsequently, the functional annotation of each deleterious variant was collected, after removing redundancy, over 3000 genes were subject to gene ontology (GO) enrichment analysis (Table S3). The results indicated that many of these genes functioned in sensory perception of smell, detection of stimulus, sensory perception of chemical stimulus, nervous system processes, carboxylic acid metabolic processes, and double-strand break repair (Table S3). 

**Figure 4:**
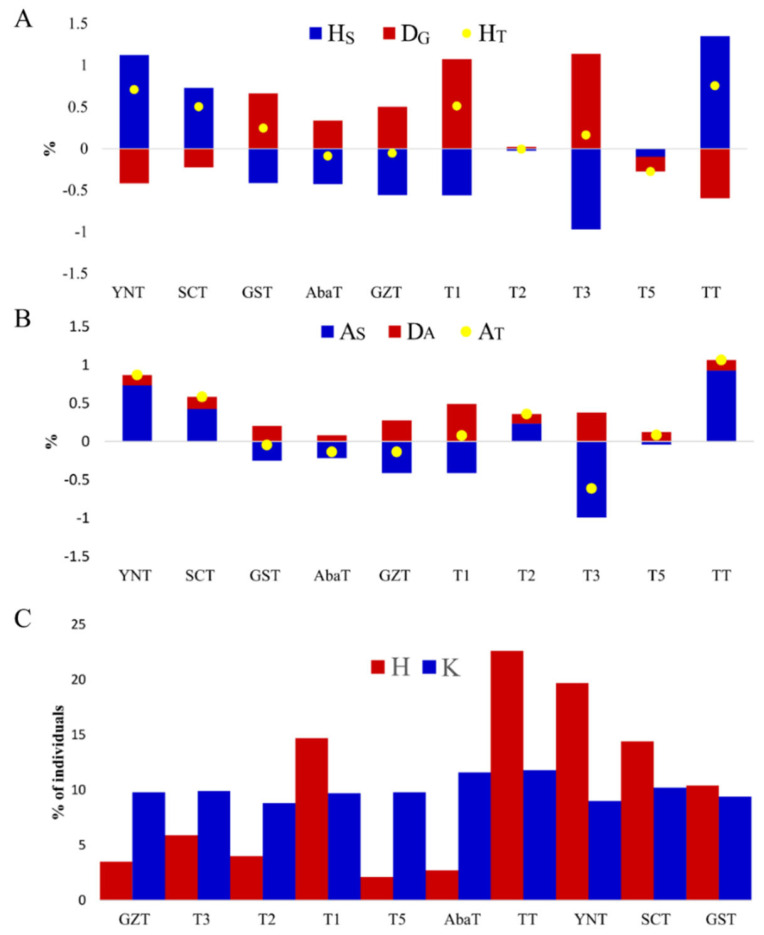
Contribution to genetic diversity of 10 Tibetan pig populations. Contribution to gene diversity (**A**) and allele diversity (**B**) H_S_ and A_S_ indicate within-population gene and allele diversity, respectively. D_G_ and D_A_ indicate between-population gene and allele diversity, respectively. H_T_ and A_T_ indicate total gene and allele diversity, respectively. Contribution of individuals from each population to a pool with maximal gene diversity (H) and allele diversity (K) (**C**).

To realize the adaptive potential of populations to new environmental challenges, the maintenance of genetic diversity is a priority for conservation management [24]. Genetic diversity is usually estimated by expected heterozygosity under Hardy–Weinberg equilibrium and by allele diversity [21]. These methods are complementary since heterozygosity is sensitive to selection effects [25] and allelic diversity is sensitive to bottleneck effects [26]. In this study, methods of maximizing globally expected heterozygosity and allelic diversity were employed to compute the contribution of each population. The contribution of each population was estimated by removing a subpopulation from the meta-population and calculating the change in gene diversity and allelic diversity [27,28]. A positive contribution value represents the loss of diversity after removing a subpopulation, while a negative value means a gain of diversity, as described by Metapop2 [18]. As shown in Figure 4, the rank of contribution to total diversity among populations was roughly the same for total heterozygosity and total allelic diversity. Removal of the TT population revealed the largest loss to total heterozygosity, H_T,_ (0.7528%), followed by YNT (0.7056%) (Figure 4A). Similarly, removal of the TT population revealed the largest loss to total allelic diversity, A_T,_ (1.0644%), followed by YNT (0.8696%). A gain of H_T_ was the largest in the T5 population (−0.2737%), while the gain of A_T_ was the largest in the T3 population (−0.6132%). T3 had the greatest negative contribution to both H_S_ (−0.9713%) and A_S_ (−0.9923%), implying this population had low within-population diversity (Figure 4A, B). This could be explained by its low allelic richness (1.4769) and the highest average co-ancestry (0.8128) (Table 1). The TT population presented the largest positive contribution to both H_S_ (1.3501%) and A_S_ (0.9260%), suggesting this population had the highest within-population diversity. This also could be explained by its high allelic richness (1.6028) and low average co-ancestry (0.7409) (Table 1). In conservation programs, generally maximizing expected heterozygosity is equivalent to minimizing average co-ancestry within populations [29]. However, the TT population had the lowest negative contribution to D_G_ (−0.5973%) as its average Nei’s distance against others (0.1881) was the smallest among the populations (Figure 2). The TT population contributed positively to D_A_, which is derived from its high A_R_ (1.6028) (Table 1). This explains why its removal brings a loss in between-population gene diversity but a gain in between-population allelic diversity. Optimum contributions of each subpopulation to the meta-population pool were demonstrated based on maximal expected heterozygosity and total number of alleles (Figure 4C). Expectedly, the TT population made the largest contribution to gene diversity (H) and allele diversity (K). For gene diversity, the TT, YNT, T1, SCT, and GST populations contributed more than 10%, to allele diversity, TT, AbaT and SCT contributed more than 10% while the others accounted for ~9% of the total. In aggregate, these data suggest that a conservation plan for the Tibetan pig would at least initially start by focusing on protecting the TT population. 

### 3.4. Selective Sweep

**Figure 5:**
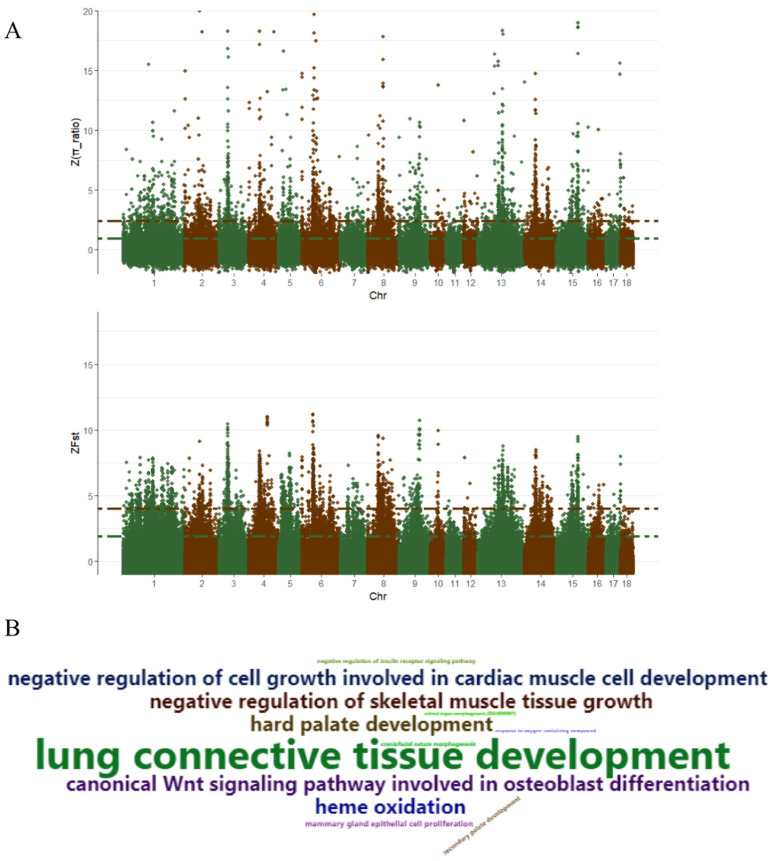
Genome regions of selective signature and GO enrichment analysis. Distribution of Z-*F*_ST_ and Z-*θ*_π_ ratio (*θ*_π,wild_/*θ*_π,Tibetan_) (**A**). Each dot represents a 10 kb non-overlapping window, red and green lines represent the 95th and 99th quantile, respectively. Wordcloud plot showed the interesting GO enrichment terms (**B**).

Tibetan pigs originated on the Tibetan Plateau, and have undergone long term natural selection in that harsh environment [5]. Here, in order to investigate the effects of domestication and selection on the Tibetan pig genome, we performed a selective sweep analysis of the whole genome. Since the extent of LD (Linkage disequlibrium) is less than 10 kb in Chinese domestic pigs, we set the selective windows size at 10 kb [1,30]. Over 220,000 non-overlapping windows were scanned for selective signatures. Selective signatures were identified in 863 windows by combining Z-*F*_ST_ (Top 1%) and, Z-θ_π_ ratio (θ_π,wild_/θ_π,Tibetan_, Top 1%) (Figure 5A). After functional analysis of selective signatures, 384 genes were identified (Table S4). Results from GO analysis identified genes involved in lung connective tissue development and lung development, hard palate development and craniofacial suture morphogenesis, sensory perception of smell, negative regulation of cell growth involved in cardiac muscle cell development, response to oxygen-containing compound, growth hormone secretion and animal organ morphogenesis. (Figure 5B and Table S5).

In this study, we focused on some of the difference between wild and Tibetan pigs. These included facial appearance, coat color, hormone metabolism, growth, and hypoxia adaptation-related processes. Three genes, *FZD1*, *DLG,1* and *MMP25*, were involved in hard palate development and craniofacial suture morphogenesis. FZD1 and its receptor are expressed ventral to the telencephalon and in periocular mesenchyme during the development of the face [31]. Loss of function variants in *DLG1* are associated with non-syndromic discontinuous cleft lip and palate [32]. *MMP25* expression appears to have a crucial role throughout all stages of palatal development [33]. These genes may have played an important role in face or palatal changes during domestication of Tibetan pig. A peak of selective signal (Z-*F_ST_* 7.69 and Z-*θ*_π_ ratio 318.74) was identified in the region Chr 6:180001:190000. The *MC1R* gene is located in this selective region, which is reported to contribute to the black color of Chinese domestic pig [34]. *TBX19* is involved in the selection effect of domestication on the pig genome [35]. *TBX19* plays a crucial role in activating pituitary cell differentiation and induces proopiomelanocortin expression, which leads to generating the adrenocorticotropic hormone working in the hypothalamic–pituitary–adrenal axis [35,36]. Five cytochrome P450 genes, *CYP2F1*, *CYP2A19*, *CYP2B22*, *CYP2B22*, and *CYP2S1,* and *ENSSSCG00000028627*, and a lncRNA ENSSSCG00000041224 are located in a 120 kb selective region. *ENSSSCG00000028627* is located in the peak of selective signal (Z-*F_ST_* 8.15and Z-*θ*_π_ ratio 4.23). These genes function in the synthesis of steroids, cholesterol and other lipids [37]. It suggests that hormone metabolic processes were likely under positive selection in Tibetan pigs. Unexpectedly, *MSTN* and *ESR1* were selected in the Tibetan pig in relative to wild pig. *MSTN*, a interesting gene, negatively regulates skeletal muscle cell proliferation and differentiation. Mutation in *MSTN* is associated with muscle hypertrophy [38]. *MSTN* was selected in lowland domestic pigs relative to wild pig [35]. *ESR1* was also selected in Chinese domestic pigs relative to wild pigs [39], participating in the reproduction process [40]. Long-standing trade likely introduced the lowland domestic pig to Tibetan plateau where they interbred with the Tibetan pig [4]. The *MSTN* and *ESR1* genes might be the result of gene flow from lowland pigs to Tibetan pigs. There were also three genes participating in sensory perception of smell, *OR1F1*, *ENSSSCG00000037040,* and *ENSSSCG00000034150*. OR1F1, an olfactory receptor, was confirmed to function in odor perception activation [41]. Previous publications also demonstrated Tibetan pigs lost some olfactory receptor genes across the genome [7,42]. 

Some interesting genes involved in lung connective tissue development and lung development included *RXFP1*, *CIC*, *MAPK8IP3*, *CCDC39,* and *SPRY1* (Table S4). *RXFP1* is directly associated with pulmonary function; low expression of this gene is found in diseased lungs [43]. *RXFP1*, a relaxin family peptide receptor, protects astrocytes from hypoxia [44]. *HIF3A* and *EGLN2* were selected in Tibetan pigs. These genes are involved in the hypoxia pathway in Tibetan cattle [45]. Unexpectedly, relatively fewer hypoxia adaptation related genes were identified in selective sweep analysis. After literature mining, we found that *EPAS1* is considered a crucial gene in hypoxia adaptation of Tibetan pig [1,4,7]. However, no significant selective signal was identified near the *EPAS1* gene in this study. These previous studies aimed to find the candidate genes involved in hypoxia adaptation of Tibetan pig and used Chinese lowland native pigs as the control [1,4,7], This study, however, aimed to identify the domestication region of the Tibetan pig genome, and so used wild pigs as the control. What we found were many phenotype-related genes, including coat color, facial appearance, sensory perception of smell, growth, and hormone metabolism.

Additionally, the integrated analysis identified three potential deleterious genes selected in Tibetan pigs relative to wild pigs. *DUOX2* is a transmembrane protein located at the surface of thyroid follicular cells. Mutation in this gene is associated with congenital hypothyroidism [46]. *SULT1B1* is a protein gene involved in cytochrome P450 metabolism pathway [37]. *SCAPER* mutation causes autosomal recessive retinitis pigmentosa with intellectual disability [47]. These data suggest that domestication had negative, as well as positive, effects on the genome of Tibetan pig. 

## 4. Conclusions

Based on population genetic analysis, the Tibetan pigs living in various regions of the Tibetan Plateau have undergone slight genetic differentiation. Analysis of the distribution of deleterious variants indicated that population-specific deleterious variants accounted for the major component of loci with negative effects. Results from contribution to genetic diversity analysis demonstrated that the TT population made the highest contribution to gene diversity and allele diversity to the meta-population. Selective sweep analysis revealed that numerous genes, including *RXFP1*, *FZD1*, *OR1F1*, *TBX19*, *MSTN*, *ESR1, MC1R*, *HIF3A*, and *EGLN2* are involved in hypoxia adaptation, coat color, hormone metabolism, facial appearance, and perception of smell. These findings help us understand the origin and domestication of the Tibetan pigs, as well as optimizing conservation programs.

## Figures and Tables

**Table 1:** Genetic diversity in 10 Tibetan pig populations.

Population	*n*	Origin	*H_O_*	*H_E_*	*A_R_*	*F*	*f*_ii_
YNT	6	Qinghai-Tibet Plateau, Yunnan province	0.3728	0.3714	1.5902	0.0083	0.7456
SCT	6	Qinghai-Tibet Plateau, Sichuan province	0.3594	0.3760	1.5700	0.0514	0.7539
GST	4	Qinghai-Tibet Plateau, Gansu province	0.4385	0.4151	1.5256	0.0189	0.7828
AbaT	4	Qinghai-Tibet Plateau, Tibetan Qiang Autonomous Prefecture of Ngawa	0.3262	0.4178	1.5279	0.2194	0.7832
GZT	5	Qinghai-Tibet Plateau, Tibetan Autonomous Prefecture of Garzê	0.2671	0.3951	1.5150	0.3240	0.7836
TT	6	Qinghai-Tibet Plateau, Tibet	0.3324	0.3670	1.6028	0.0987	0.7409
T1	5	Qinghai-Tibet Plateau	0.2872	0.3956	1.5151	0.2742	0.7835
T2	5	Qinghai-Tibet Plateau	0.2569	0.3874	1.5574	0.3399	0.7698
T3	3	Qinghai-Tibet Plateau	0.4036	0.4682	1.4769	0.1384	0.8128
T5	6	Qinghai-Tibet Plateau	0.2379	0.3681	1.5394	0.3537	0.7712

Note: *H_O_*, observed heterozygosity; *H_E_*, expected heterozygosity; *A_R_*, allelic richness; *F*, inbreeding coefficient. *f*_ii_, Average coancestry within the population.

## Supplementary Materials

The following are available online at https://www.mdpi.com/2076-2615/10/8/1297/s1, Table S1: Sample information of 10 Tibetan pig populations, Table S2: Annotation information and frequency distribution of deleterious variants of Tibetan pigs, Table S3: GO enrichment analysis of deleterious variants, Table S4: Gene list of selective regions, Table S5: GO enrichment analysis of genes under selection.
